# Efforts of Nature to Form a Pupil at That Point of the Eye Where It Will Be Most Useful

**Published:** 1844-09

**Authors:** 


*Efforts of nature to form a pupil at that point of the eye where it will be most useful.*—Mr. J. B. Estlin, of Bristol, states that in examining eyes where central opacity of the cornea existed, he has been repeatedly struck with the appearance of the pupil, which he has observed to be of a long, narrow shape, as if the longitudinal fibres of the iris had been split for the purpose of producing an aperture exactly opposite the clear portion of cornea, so as to be in the most suitable part for distinct vision. He was inclined to attribute this to some accidental circumstance; and when it existed in persons in whom the opacity of the cornea was occasioned by external injury, he supposed it to depend upon the fortunate coincidenc of a wound on that part of the iris at the time when the original injury was inflicted. But having remarked, with much interest, in operations for artificial pupil, that an aperture in the iris, made at some little distance from the clearest portion of cornea, will in time extend itself, so as to be exactly in the position he at first designed and wished it to be, he could have no doubt that occasionally, in other cases of opacity from disease, nature endeavors to remedy the evil by a similar effort.—*Prov. Med. Jour.*—*Am. Jour. Med. Sci.*

